# Generation of whole genome sequences of new *Cryptosporidium hominis* and *Cryptosporidium parvum* isolates directly from stool samples

**DOI:** 10.1186/s12864-015-1805-9

**Published:** 2015-08-29

**Authors:** Stephen J. Hadfield, Justin A. Pachebat, Martin T. Swain, Guy Robinson, Simon JS Cameron, Jenna Alexander, Matthew J. Hegarty, Kristin Elwin, Rachel M. Chalmers

**Affiliations:** Cryptosporidium Reference Unit, Public Health Wales Microbiology ABM, Singleton Hospital, Sgeti, Swansea, SA2 8QA United Kingdom; Institute of Biological, Environmental & Rural Sciences, Aberystwyth University, Penglais, Aberystwyth, Ceredigion SY23 3DA United Kingdom

**Keywords:** Whole genome sequencing, Illumina, Nextera XT, Cryptosporidium, Stool, Immunomagnetic separation

## Abstract

**Background:**

Whole genome sequencing (WGS) of *Cryptosporidium* spp. has previously relied on propagation of the parasite in animals to generate enough oocysts from which to extract DNA of sufficient quantity and purity for analysis. We have developed and validated a method for preparation of genomic *Cryptosporidium* DNA suitable for WGS directly from human stool samples and used it to generate 10 high-quality whole *Cryptosporidium* genome assemblies. Our method uses a combination of salt flotation, immunomagnetic separation (IMS), and surface sterilisation of oocysts prior to DNA extraction, with subsequent use of the transposome-based Nextera XT kit to generate libraries for sequencing on Illumina platforms. IMS was found to be superior to caesium chloride density centrifugation for purification of oocysts from small volume stool samples and for reducing levels of contaminant DNA.

**Results:**

The IMS-based method was used initially to sequence whole genomes of *Cryptosporidium hominis gp60* subtype IbA10G2 and *Cryptosporidium parvum gp60* subtype IIaA19G1R2 from small amounts of stool left over from diagnostic testing of clinical cases of cryptosporidiosis. The *C. parvum* isolate was sequenced to a mean depth of 51.8X with reads covering 100 % of the bases of the *C. parvum* Iowa II reference genome (Bioproject PRJNA 15586), while the *C. hominis* isolate was sequenced to a mean depth of 34.7X with reads covering 98 % of the bases of the *C. hominis* TU502 v1 reference genome (Bioproject PRJNA 15585).

The method was then applied to a further 17 stools, successfully generating another eight new whole genome sequences, of which two were *C. hominis* (*gp60* subtypes IbA10G2 and IaA14R3) and six *C. parvum* (*gp60* subtypes IIaA15G2R1 from three samples, and one each of IIaA17G1R1, IIaA18G2R1, and IIdA22G1), demonstrating the utility of this method to sequence *Cryptosporidium* genomes directly from clinical samples. This development is especially important as it reduces the requirement to propagate *Cryptosporidium* oocysts in animal models prior to genome sequencing.

**Conclusion:**

This represents the first report of high-quality whole genome sequencing of *Cryptosporidium* isolates prepared directly from human stool samples.

## Background

*Cryptosporidium* is a genus of protozoan parasites which are a major cause of gastrointestinal infection worldwide. Symptoms of human cryptosporidiosis include diarrhoea abdominal pain, nausea or vomiting and low grade fever. These are usually self-limiting, albeit after 2 or even 3 weeks, but can be prolonged or invasive and life-threatening in patients with severe T-cell immune-deficiency [1]. *Cryptosporidium* spp. are also increasingly recognised as an important cause of childhood morbidity and mortality in developing countries [2]. Treatment options are limited, with only one specific licenced therapeutic. Of the 26 *Cryptosporidium* spp. currently named, *Cryptosporidium hominis* and *Cryptosporidium parvum* are the most common causes of cryptosporidiosis in humans globally. Research into the biology of the organism has been partly hampered by the lack of an effective cell culture system for propagation and the limited availability of genomic sequence data, impacting, for example, on progress in understanding the taxonomic position of the protozoan, therapeutic drug discovery, and identification of diagnostic, virulence and subtyping markers.

Whole genome sequences produced using Sanger sequencing techniques, are publically available for single isolates of three species which have historically provided reference genomes for the mainly anthroponotic *C. hominis* (isolate TU502), the zoonotic *C. parvum* (isolate IOWA II), and *C. muris* (isolate RN66) which primarily infects rodents [3, 4]; NCBI BioProjects: PRJNA15585 PRJNA15586, PRJNA32283). Recently, new whole genome sequences have been made available from next generation sequencing (NGS) platforms, providing updated versions of the genome sequences for *C. hominis* TU502 and *C. parvum* IOWA II and new genome sequences for additional isolates: C. *hominis* UKH1, *C. meleagridis* UKMEL1 and *C. baileyi* TAMU-09Q1 (www.cryptoDB.org). The powerful technologies provided by NGS have dramatically reduced the cost and time required for whole genome sequencing, greatly increasing our knowledge of *Cryptosporidium* spp. by, for example, allowing in-depth, high-resolution comparison of isolates and deep sequencing of selected loci for investigation of parasite population variability. However, significant challenges exist for generation of whole genome sequence data, largely because even the most sensitive sample preparation kit for the generation of sequencing libraries demands at least 1 ng genomic DNA; *C. parvum* oocysts each contain 40 fg DNA [5] thus requiring an estimated 2.5 × 10^4^ highly-purified oocysts. Human stool samples received at diagnostic laboratories tend to be small in volume and contain relatively small numbers of *Cryptosporidium* oocysts amongst very large numbers of non-target organisms, mainly bacteria, which is problematic for the production of the purity of genomic DNA required for analysis. Whole genome sequences generated to date have either utilised experimental animal infections for propagation of sufficient material [6] or obtained and processed large volumes of animal faeces from natural infections [7]; both approaches restrict the information available to a small number of sufficiently abundant isolates and may introduce the potential for strain selection bias. The former is most especially unsustainable for large-scale investigations for practical, financial and ethical reasons, particularly for *C. hominis* which has no symptomatic animal model and requires passage in gnotobiotic piglets [8] or immunosuppressed Mongolian gerbils [9]. Enrichment of genomic *Cryptosporidium* DNA for WGS from stools has recently been described using whole genome amplification but significant contamination was reported [10].

Here we describe the development and validation of a protocol for preparation of genomic *Cryptosporidium* DNA from routinely submitted diagnostic human stool samples suitable for whole genome sequencing. The method has the following characteristics which specifically address the challenges described above: enhanced purification of oocysts degradation of non-*Cryptosporidium* contaminants, and use of an NGS library preparation kit capable of processing as little as 1 ng of genomic DNA.

## Results and discussion

### Characterisation of isolates prior to WGS

Characterisation by *C. parvum* and *C. hominis*-specific real-time polymerase chain reaction (PCR) revealed no evidence of the presence of more than one species in any of the stools tested. Only the expected species were identified by Sanger sequence analysis of the 18S rRNA actin, *hsp70* and *COWP* genes. *Gp60* subtypes were identified as shown in Tables 1 and 2. There was no evidence from the sequencing chromatograms that any of the isolates were of mixed populations of *Cryptosporidium* species or *gp60* subtypes.

**Table 1:** Properties of *Cryptosporidium* oocyst and DNA preparations from two pilot phase stool samples

Isolate, species and gp60 subtype	Preparation stage	qPCR 18S rRNA gene (C_T_) from original stool sample	Quantification of *Cryptosporidium* processed by IMS		Bacterial DNA calculated from qPCR 16 s rRNA gene (ng) (% reduction compared with salt-floated suspension)	Total DNA from Qubit (ng)
			Numbers of oocysts counted; genome copies derived from microscopy counts	Genome copies calculated from qPCR 18 s rRNA gene		
UKP2, *C. parvum* IIaA19G1R2	Salt-floated suspension	28.8	1.0 ×10^6^; 4.0×10^6^	4.27×10^5^	323.1	ND
	Post IMS, bleach treated suspension		3.7×10^5^; 1.5×10^6^	2.99×10^5^	4.2 (98.7 % reduction)	2.44
	Post CsCl, bleach treated suspension		1.3×10^5^; 5.2 ×10^5^	1.99×10^5^	ND^a^	1.52
UKH3, *C. hominis* IbA10G2	Initial suspension	26.7	1.0×10^6^; 4.0×10^6^	4.6×10^5^	86.7	ND
	Post IMS, bleach treated suspension		2.9×10^5^; 1.2×10^6^	1.3×10^6^	4.4 (94.9 % reduction)	2.68

*ND*, not done

^a^Not tested in order to preserve DNA as total DNA level was low

**Table 2:** Assessment of *Cryptosporidium* preparation from 17 stool samples containing *C. hominis* or *C. parvum* oocysts

		Initial suspension prior to processing by IMS			DNA extracted from IMS purified oocysts			
Sample	Species and *gp60* subtype	qPCR 18S rRNA C_T_	Number of oocysts counted	Visual condition of oocysts	qPCR 18S rRNA C_T_	Calculated genome copies	Mass of DNA (ng)	>1 ng DNA available for library preparation (isolate reference number)
1	*C. hominis* IaA14R3	ND	1.9×10^7^	Good	17.7	5.53×10^7^	77	Yes (UKH4)
2	*C. hominis* IbA10G2	23.5	1.6×10^7^	Good	20.0	1.24×10^7^	49.55	Yes
								(UKH5)
3	*C. hominis* IdA18	29.3	2.1×10^5^	Good	25.7	1.64×10^5^	Below threshold	No
4	*C. parvum* IIaA15G1R1	31.6	7.5×10^4^	Good	25.9	1.49×10^5^	0.00	No
5	*C. parvum* IIaA15G1R2	31.1	5.0×10^4^	Good	25.1	2.36×10^5^	0.00	No
6	*C. parvum* IIaA15G2R1	28.1	3.6×10^5^	OK - a few empty oocysts, didn't stain well	28.6	2.66×10^4^	0.00	No
7	*C. parvum* IIaA18G2R1	29.1	1.5×10^6^	Good	22.2	2.99×10^6^	5.45	Yes
								(UKP3)
8	*C. parvum* IIaA15G2R1	25.5	2.8×10^6^	Good	20.1	5.99×10^6^	18.65	Yes
								(UKP4)
9	*C. parvum* IIaA15G2R1	27.5	9.1×10^5^	Good	23.2	1.52×10^6^	2.68	Yes
								(UKP5)
10	*C. parvum* IIaA15G2R1	27.1	2.7×10^6^	Good	23.4	1.32×10^6^	4.40	Yes
								(UKP6)
11	*C. parvum* IIaA17G1R1	23.8	9.0×10^6^	Good	20.3	1.01×10^7^	28.65	Yes
								(UKP7)
12	*C. parvum* IIaA19G2R1	30.4	1.1×10^6^	Good	24.4	3.74×10^5^	0.8	No
13	*C. parvum* IIaA19G2R1	29.2	1.1×10^6^	Good	28.7	2.53×10^4^	0.00	No
14	*C. parvum* IIaA19G2R1	30.6	5.2×10^5^	OK	31.8	3.58×10^3^	0.00	No
15	*C. parvum* IIaA19G2R1	34.0	3.9×10^4^	OK - a few empty oocysts	No amplicon detected	0	ND	No
16	*C. parvum* IIdA22G1	28.4	2.3×10^6^	Good	21.9	3.62×10^6^	6.55	Yes
								(UKP8)
17	*C. parvum* IIdA24G1	30.9	1.2×10^6^	Good	23.9	1.01×10^6^	2.88	Yes^a^

^a^A technical problem resulted in no data being produced for this sample

### Evaluation of oocyst preparation methods in the pilot phase

Salt-floated suspensions of 1 × 10^6^*C. parvum* and *C. hominis* oocysts yielded after IMS, 6.1 × 10^5^ (61 % recovery) and 4.5 × 10^5^ oocysts (45 % recovery, respectively. After bleach-treatment, 3.7 × 10^5^*C. parvum* and 2.9 × 10^5^*C. hominis* oocysts were counted, sufficient for DNA extraction.

Caesium Chloride (CsCl) density gradient centrifugation of the same original number of oocysts in salt-floated suspension yielded 2×10^5^*C. parvum* oocysts (20 % recovery) and <2 × 10^4^*C. hominis* oocysts (<2 % recovery undetectable in counting chamber) and after bleach treatment 1.3 × 10^5^ and <5.3 × 10^3^ (undetectable), respectively, indicating that IMS was the more efficient method for purification of oocysts derived from small volume stool samples (Table 1). Due to undetectable numbers of oocysts in the CsCl-prepared *C. hominis* suspension, this was not processed further. Although CsCl has been used successfully for oocyst preparation from bulk samples collected repeatedly from a naturally infected animal [7], the collection of bulk or multiple repeat samples is not practical for stool collection from most human patients.

The use of quantitative PCR (qPCR) to derive oocyst numbers in the salt-floated suspensions resulted in estimates 9.4-fold (*C. parvum*) and 8.6-fold (*C. hominis*) lower than those derived from microscopic counts of intact oocysts in good condition (Table 1). Either *Cryptosporidium* DNA was lost during the extraction process or there was uncertainty of the measurements. The presence of PCR inhibitors co-extracted with the DNA would be expected to lead to an underestimation of DNA concentration by qPCR in these stool-derived samples when compared to standard curves derived from cloned template. Oocyst numbers derived from qPCR of DNA extracted from the more highly-purified IMS and CsCl suspensions were closer to actual microscopic counts than those for the salt-floated suspensions (Table 1); it is likely that DNA from more highly-purified oocysts was more closely represented by the standard curve plasmid DNA and therefore more accurately quantified than that prepared from the salt-floated suspensions.

A substantial decrease in the mass of bacterial DNA was identified after both IMS and bleach-treatment (Table 1) reduced by 94.9 % (*C. hominis*) and 98.7 % (*C. parvum*) compared with the salt-floated suspensions. CsCl-prepared *C. hominis* DNA was not processed for library preparation due to its low total DNA concentration (see below). Total DNA concentration in the DNA extracts from the *C. parvum* IMS and CsCl-prepared samples and the *C. hominis* IMS-prepared sample, measured by Qubit fluorometric quantitation, was >1 ng and therefore sufficient for NGS library preparation.

These measurements highlighted the utility of the IMS and bleaching processes for reduction of extraneous DNA without significantly affecting the amount of *Cryptosporidium* DNA. Seth-Smith et al. also used IMS to reduce non-target organisms prior to *Chlamydia trachomatis* DNA extraction [11] however, in the case of *Cryptosporidium*, the oocyst wall affords the opportunity to further reduce bacterial contamination with brief sodium hypochlorite treatment without damaging the target DNA, whilst providing challenges with regard to fracturing the oocyst wall prior to DNA extraction when dealing with low numbers of oocysts.

### Evaluation of DNA preparation in the main phase

**Fig. 1:**
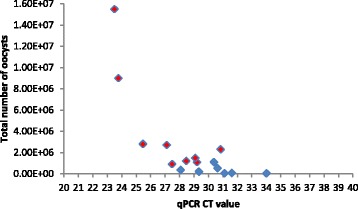
Relationship between total oocyst numbers, C_T_ values and concentration of recovered DNA. Oocyst numbers (counted by microscopy), and C_T_ values in the salt-floated suspension were determined prior to processing, and compared to final DNA recovery. Red, WGS successful; Blue, <1 ng DNA available (below manufacturer’s threshold for library preparation)

**Fig. 2:**
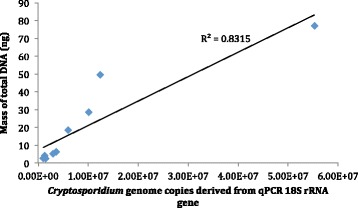
Relationship between *Cryptosporidium* genome copies and total DNA in extracts prior to whole genome sequencing

Of the 17 stools processed nine produced sufficient total DNA for library preparation, of which two contained *C. hominis* and seven *C. parvum*. The median low threshold cycle (C_T_) value for suspensions that generated sufficient DNA for whole genome sequencing (27.3, range 23.5-30.9) was significantly lower than for those that did not (30.5, range 28.1-34.0) (p = 0.009). Although there was some overlap in C_T_ values, suspensions containing oocysts that were in good condition were more likely to produce whole genome sequences (Table 2). Although qPCR was not a reliable indicator of oocyst abundance in salt floated suspensions in the pilot phase, the combination of qPCR, oocyst counts and visual condition proved to be a good and practical predictor of suitability of salt-floated suspensions for further processing in the main phase (Table 2 Fig. 1). The importance of microscopic examination is notable because although oocyst counts are time consuming so is IMS processing (which is also expensive), and pre-screening in this way would maximise use of resources in larger scale applications. Post IMS there was clear delineation of C_T_ values between sufficient and insufficient preparations the median C_T_ value for those that generated sufficient DNA for whole genome sequencing (21.9 range 17.7-23.9) was significantly lower than those that did not (25.9, range 24.4-31.8) (p = 0.0009). There was a significant relationship between *Cryptosporidium* 18S rRNA gene qPCR and mass of DNA in the final extracts (R^2^ = 8.315, see Fig. 2) indicating this comprised a sufficiently large proportion of *Cryptosporidium* DNA. Although 16S rRNA gene qPCR had proved useful in the pilot phase to demonstrate reduction in bacterial DNA, it was not used in the main phase. *Cryptosporidium* 18S rRNA gene qPCR was evidently more useful in process monitoring, with the pilot phase results showing that *Cryptosporidium* genome copies could more accurately be derived from these data, with the additional benefit of reduced analysis preserving material for downstream use.

### Results of next generation sequencing

Including both pilot and main phases a total of three *C. hominis* and seven *C. parvum* whole genomes were successfully sequenced, reads aligned to reference genomes, and contigs *de novo* assembled and annotated, with an additional sequence generated via the CsCl method for the *C. parvum* isolate in the pilot phase.

In the pilot phase sequencing *C. parvum* UKP2 using the Illumina MiSeq achieved 100 % coverage of the *C. parvum* IOWA II reference genome at a mean 51.80X depth using the IMS-based method, 100 % coverage at a mean 46.84X depth using the CsCl-based method, and for *C. hominis* UKH3 98 % coverage of the *C. hominis* TU502 reference genome at a mean 34.71X depth (Table 3). When aligned to the reference genomes, 90.30 % of reads from *C. hominis* UKH3, 92.51 % of reads from *C. parvum* UKP2 IMS preparation and 81.86 % reads from *C. parvum* UKP2 CsCl preparation mapped to the reference sequences the percentage of unmapped reads were likely from residual bacterial contamination (although this appears to be low) and sequencing errors and was greater for the CsCl preparation compared to the IMS preparations.

**Table 3:** Quality of whole genome sequences generated directly from stools containing *C. hominis* and *C. parvum*

Isolate; species and *gp60* subtype; BioProject number	Total # base pairs sequenced (after merging reads)	Total # base pairs mapped to reference sequence	Proportion of *Cryptosporidium* DNA compared to background	% of reference genome covered	Mean depth on reference sequence	% AT content of reads mapped to reference sequence
Pilot phase						
UKH3 (IMS preparation)	337,791,948	305,024,423^a^	90.30 %	98 %	34.71 ×	67.49 %
* C. hominis* IbA10G2						
PRJNA253834						
UKP2 (IMS preparation)	510,081,295	471,881,392^b^	92.51 %	100 %	51.80 ×	67.92 %
* C. parvum* IIaA19G1R2						
PRJNA253836						
UKP2 (CsCl preparation)	521,277,984	426,692,177^b^	81.86 %	100 %	46.84 ×	67.85 %
* C. parvum* IIaA19G1R2						
PRJNA253836						
Main phase						
UKH4	2,164,426,378	1,828,866,488^a^	84.50 %	96 %	209.17 ×	62.72 %
* C. hominis* IaA14R3						
PRJNA253838						
UKH5	2,182,317,271	1,765,458,438^a^	80.90 %	96 %	201.92 ×	63.03 %
* C. hominis* IbA10G2						
PRJNA253839						
UKP3	1,703,132,267	1,514,828,932^b^	88.94 %	99 %	166.42 ×	63.55 %
* C. parvum* IIaA18G2R1						
PRJNA253840						
UKP4	1,967,147,533	1,751,979,030^b^	89.06 %	99 %	192.48 ×	63.60 %
* C. parvum* IIaA15G2R1						
PRJNA253843						
UKP5	288,922,509	244,528,063^b^	84.63 %	99 %	26.86 ×	67.67 %
* C. parvum* IIaA15G2R1						
PRJNA253845						
UKP6	1,169,379,989	954,176,437^b^	81.60 %	99 %	104.83 ×	67.73 %
* C. parvum* IIaA15G2R1						
PRJNA253846						
UKP7	795,715,168	708,613,859^b^	89.05 %	99 %	77.85 ×	63.64 %
* C. parvum* IIaA17G1R1						
PRJNA253847						
UKP8	1,896,616,473	1,587,380,412^b^	83.70 %	98 %	174.39 ×	63.20 %
* C. parvum* IIdA22G1						
PRJNA253848						

^a^mapped against *C. hominis* TU502 v1 reference sequence (Bioproject PRJNA15585)

^b^mapped against the *C. parvum* IOWA II v1 reference sequence (Bioproject PRJNA15586) using default mapping conditions

In the main phase eight of the nine DNA extracts generated high-quality data using the Illumina HiSeq the proportion of reads mapping to the reference sequence ranged from 80.90 % to 89.06 % with 0.96 to 0.99 fraction of the reference genome covered confirming the suitability of the Illumina Nextera XT sample preparation for generation of *Cryptosporidium* genomes from clinical isolates (Table 3). One sample failed to produce sufficient data probably because insufficient library DNA was added at the pooling stage.

The post-assembly genome improvement protocol (PAGIT) [12] improved assemblies produced from the sequenced reads were of high-quality and statistics describing them are given in Table 4. Of the 10 assemblies 8 could be assembled into less than 70 scaffolds, with the assembly N50 metric equal to just over 1 Mb for most assemblies (i.e. 50 % of the genome has been assembled into scaffolds with a minimum length of 1 Mb). Using the Bowtie2 read aligner [13] with the default settings we were able to align between 82.0 % and 96.2 % of sequenced reads as concordant pairs to the final genome assemblies, indicating that the genome assemblies are relatively complete. Rapid annotation transfer tool (RATT) [14] was able to transfer between 92.1 % and 95.9 % of the 3,805 gene annotations from *C. parvum* Iowa II to the assemblies thus providing a sound basis for further studies into sequence and structural variation. Although notably greater depth of coverage was achieved with the Illumina HiSeq (26.9-209.2 X) than with the MiSeq, the MiSeq genome assemblies were nonetheless of equivalent quality: see the statistics for UKH3 and UKP2 in Table 4.

**Table 4:** Summary of improvements made to the initial genome assemblies by PAGIT

Isolate	Initial assembly statistics: No. N50 Av. (kb)	Gaps closed by IMAGE	ICORN Sequence corrections: SNP Indel	RATT Gene annotations transferred	Final assembly statistics: No. N50 Av. Size (kb)	Paired reads aligning concordantly (%)	Contaminant sequence NC BCT (kb)
UKH3	1902 94.7 4.9	330	210 27	3649	34 1000 268.7 9136	87.5	41.1 0.0
UKH4	2375 29.1 4.0	343	584 99	3503	110 1032 84.4 9293	93.5	40.1 14.1
UKH5	1305 29.9 7.0	437	189 61	3569	65 1006 142.4 9257	96.2	6.9 2.4
UKP2	3084 105.2 3.1	174	182 48	3600	67 1011 136.7 9164	85.1	89.6 1.6
UKP3	919 57.5 10.0	259	226 60	3546	55 1009 167.7 9224	90.2	2.0 0.0
UKP4	1966 49.3 4.8	334	170 108	3553	52 891 177.4 9222	91.0	7.3 0.7
UKP5	3830 9.7 2.4	1435	341 80	3527	47 1031 198.4 9329	88.6	31.4 14.6
UKP6	16895 40.3 0.8	274	3144 394	3565	417 1014 22.7 9471	81.2	685.7 69.5
UKP7	1158 30.2 7.8	321	185 80	3556	55 1026 168.7 9278	90.4	2.5 0.0
UKP8	1792 42.0 5.2	356	152 47	3545	63 1015 147.4 9290	93.5	37.9 24.4

The assembly statistics (initial and final) include the number of scaffolds, scaffold N50 metric, scaffold mean length (Av.), and the total size of the final assembly. Gene annotations were transferred by RATT out of a total of 3805 gene annotations in the reference assembly. The “Contaminant sequence” column refers to the total length non-concordantly mapping read pairs that have been assembled separately and identified as non-cryptosporidium sequence (NC) and bacterial sequence (BCT)

In order to investigate the possibility of bacterial contamination for each isolate, any read pairs that did not map concordantly to the final *de novo* contig assemblies were assembled separately. From these assemblies of contaminants, contigs greater than 300 bp were blasted [15] against all *Cryptosporidium* assemblies available on the National Center for Biotechnology Information (NCBI: *C. muris* RN66, *C. parvum* Iowa II, *Cryptosporidium* sp. chipmunk LX-2015, and *C. hominis* TU502) [16]. Using a Basic Local Alignment Search Tool (BLAST) e-value < 1^−10^ potential *Cryptosporidium* sequences were filtered out of these assemblies. The remaining non-*Cryptosporidium* contigs (column “NC” in Table 4) were subsequently blasted against all bacterial genomes available on the NCBI, and using an e-value < 1^−10^ the total amount of bacterial sequence was identified (column “BCT” in Table 4). The amount of contaminating sequence was too small, too fragmented (the longest contig from any of these assemblies was 971 bp), or too low in complexity to reliably identify any particular contaminating bacteria species.

The serine tri-nucleotide repeat rich *gp60* gene sequence is used in sub-typing to study the taxonomy and transmission of the *Cryptosporidium* parasite [17, 18]. Searching for the *gp60* gene sequences from each isolate in the respective assemblies with BLASTN produced 100 % alignments with an E-value = 0.0 of 681 to 755 bp in length: although no alignments were reported over the low complexity regions at the start of the *gp60* gene because BLAST filters out such regions before aligning sequences. The 18S rRNA gene sequences were similarly found by BLAST in the assemblies with 100 % sequence identity, split into two alignments due a low complexity region in the middle of the sequence with each alignment having a length of around 250 bp to 400 bp. Exceptions were UKH4, where two copies (i.e. four alignments of 250 bp to 400 bp) of the 18S rRNA gene region could be found, all between 99.2 % to 100.0 % sequence identity, and UKH5 where there were three BLAST hits of 100.0 % sequence identity (also with 250 bp to 400 bp lengths). No evidence was found of mixed species populations. Note that five copies of the 18S rRNA gene are found in the *C. parvum* Iowa II reference genome assembly and this indicates that the assembly process used in this study has collapsed the 18S rRNA gene regions, typically into just one copy.

The genome data from this study have been deposited on the NCBI (http://www.ncbi.nlm.nih.gov/) database as individual BioProjects (Table 3) under the umbrella BioProject PRJNA215218.

## Conclusions

We have developed a method for the high-quality sequencing of *Cryptosporidium* spp. isolates using the transposome-based Nextera XT library preparation and Illumina sequencing platform directly from routinely submitted human stools left over from diagnostic testing. This has the advantage of reducing the requirement for the use of animals for *in vivo* amplification of oocysts to extract sufficient genomic *Cryptosporidium* DNA for whole genome sequencing as was used to generate the reference genomes [3, 4] and potentially extending the possibilities for more widespread investigation of *Cryptosporidium*. The generation and comparison of benchmark genomes from all *Cryptosporidium* species and genotypes would help establish more definitive taxonomy than is currently possible based on the analysis of only a tiny proportion of the genome using Sanger sequencing even of multiple loci [19]. Global surveillance and analysis of *Cryptosporidium* genomes from widespread and representative infections would be valuable for identification of possible drug targets, new diagnostic, virulence and subtyping markers.

The samples prepared in this study were chosen because of sufficient sample volume (1 to 2 ml) and adequate concentration of oocysts in the salt-floated suspension. Even so the concentration of DNA available after the purification processes was often at the limit of that acceptable for the Illumina technology used. It is evident that to extend the utility of Illumina sequencing to stools of smaller volumes or with lower concentrations of oocysts, strategies such as the application of kits requiring <1 ng DNA would be useful, as might whole genome amplification (WGA) which has proven capable of increasing genomic DNA in *Cryptosporidium* samples [20] and may be of use for producing sufficient material [10, 11].

We are currently using this technique to compare Illumina sequencing libraries generated from native and WGA-amplified DNA from clinical samples [Pachebat personal communication]. This allows the approaches to be compared and investigated for artifactual mutations that, for example, have been reported to occur at a rate of approximately 1 in 10^5^ to 10^6^ for φ29-based methods [21]. Although it required additional steps and is susceptible to contamination, WGA could expand the application of Illumina sequencing to still more samples [10].

We conclude that in our laboratory using the current Nextera XT library preparation methods the following selection criteria are valuable predictors of likely successful generation of high-quality *C. parvum* or *C. hominis* genome sequences from stool samples:

- Original stool sample volume >1 ml
- Initial salt-floated oocyst suspension 18S rRNA gene qPCR C_T_ < 30.9 AND >9×10^5^ oocysts that are in good condition with very few empty oocysts
- Post-IMS-bleach treatment 18S rRNA gene qPCR C_T_ < 24.0 and Qubit result >1 ng total DNA

However in other laboratories different factors (such as PCR efficiency) may affect measurements, and the use of different criteria may be necessary.

We have described the generation of high-quality sequences of *Cryptosporidium* directly from stool samples and assembled these into 10 high-quality draft genomes. Our method coupled with the availability of reference sequences for comparison, removes the requirement for the use of animals to passage and amplify oocyts prior to genome sequencing. It reduces the cost, and extends the potential for this technology to be applied to the rapid and accurate genomic characterisation of clinical isolates, for the purposes of control of transmission, and treatment during outbreaks.

## Methods

### Source and characterisation of *Cryptosporidium* oocysts

The work was undertaken in two phases: a pilot phase in which two stools were processed, and a main phase in which a further 17 stools were processed.

In the pilot phase of the project, two *Cryptosporidium*-positive stool samples submitted for diagnosis of diarrhoea and subsequent identification of *Cryptosporidium* spp. were selected using the criteria of volume ≥1 ml and adequate oocyst numbers indicated by low threshold cycle (C_T_) in an 18S rRNA gene real-time PCR assay [22] (Table 1). *Cryptosporidium* species were identified by Sanger sequence analysis of the ~300 bp fragment of the 18S rRNA gene and each sample confirmed as containing either *C. parvum* or *C. hominis* only using a species-specific multiplex genotyping real-time PCR targeting the *C. parvum* Lib13 and *C. hominis* A135 loci [22, 23] Cryptosporidium Reference Unit unpublished). *Gp60* subtypes were identified by sequencing ~850 bp of the *gp60* gene [18]. Additional Sanger sequence data was obtained for the actin, *hsp70* and *Cryptosporidium* oocyst wall protein (*COWP*) genes as described previously [24].

In the main phase of the project, 17 further samples meeting the stool selection criteria above, and having a range of *gp60* subtypes, were selected for purification and processing; three containing *C. hominis* and 14 *C. parvum* (Table 2).

Ethics statement: The protozoal isolates were collected as part of routine clinical service provision [25]. The study of de-identified, purified protozoal DNA and not human subjects meant that formal Human Ethics Committee approval or Informed Patient Consent was not required.

### Purification of *Cryptosporidium* oocysts

*Cryptosporidium* oocysts were harvested from 1 to 2 ml of each faecal sample using saturated salt-flotation to generate a partially purified suspension as described previously [25]. The number of oocysts in these suspensions was estimated by staining with equal volume of FITC-labelled anti-*Cryptosporidium* monoclonal antibody (Crypto-Cel, Cellabs, Australia) and counting using a Neubauer improved haemocytometer (C-Chip, Peqlab, Sarisbury Green, UK).

Oocysts were further purified from suspension by immunomagnetic separation (IMS). In the pilot phase, a total of 1×10^6^ salt-floated oocysts were made up to 10 ml with reverse osmosis (RO) water and processed through the Isolate® IMS kit (TCS Biosciences, Botolph Claydon, UK) according to the manufacturer’s instructions. Oocysts were dissociated from the magnetic beads into 1.5 ml microfuge tubes (Elkay, Basingstoke, UK), and the volume adjusted to 1 ml with RO water prior to surface sterilisation. In the main phase salt-floated oocysts were treated similarly, but all available oocysts were used (ranging from 3.9×10^4^ to 1.9×10^7^, see Table 2) and dissociated oocysts adjusted to 200 μL with RO water prior to surface sterilisation.

To investigate an alternative to IMS in the pilot phase, 1×10^6^ oocysts were also purified from the salt-floated suspensions by caesium chloride (CsCl) gradient centrifugation as described by Upton [26], the final pellets resuspended to 1 ml in RO water and oocysts enumerated by microscopy as described above prior to surface sterilisation.

### Surface sterilisation of *Cryptosporidium* oocysts

To degrade residual contaminants, each purified oocyst suspension was treated briefly with an equal volume of 0.6 % active chlorine as sodium hypochlorite (VWR, Lutterworth, UK) and washed three times with nuclease-free water by centrifugation at 1,100X *g* for 5 min using a swing rotor and soft acceleration-deceleration profile to minimise damage to oocysts. The pellets were finally resuspended in 215 μL of nuclease-free water, 15 μL used for oocyst enumeration as described above and 200 μL for DNA extraction.

### DNA preparation and characterisation

Genomic DNA was prepared from 200 μL final *Cryptosporidium* oocyst suspensions by first performing eight cycles of freezing in liquid nitrogen for 1 min and thawing at 95 °C for 1 min, and then using the QIAamp DNA extraction kit (Qiagen, Manchester, UK) according to the manufacturer’s instructions and finally eluting in 50 μL nuclease-free water. Maximum recovery tips (Axygen Inc, USA) and DNA LoBind tubes (Eppendorf AG, Germany) were used in all subsequent DNA quantification and sequencing library preparation steps.

To investigate the efficacy of *Cryptosporidium* purification in the pilot phase, bacterial, *Cryptosporidium* and total DNA concentrations were measured as described below in 50 μL samples taken from intermediate stages of the oocyst purification process and extracted as described above, as well as in the final DNA extracts. To assess suitability for NGS library preparation in the main phase of the study, all final extracts were assayed for total DNA concentrations and specific quantification of *Cryptosporidium* DNA as described below.

Estimation of bacterial load was performed by real-time quantitative (q)PCR of the 16S rRNA gene using neat genomic DNA against standards created as described by Jones et al. [27], except that a pure culture of *Escherichia coli* was used as template material. Serial dilutions of 10^0^, 10^−2^, 10^−4^, 10^−6^, 10^−8^ and 10^−10^ were used in subsequent qPCR reactions using a C100 thermal cycler (BioRad, Hercules, USA) and CFX96 optical detector (BioRad). Reactions were completed in 20 μL volumes consisting of 10 μL of SsoAdvanced SYBR Green Supermix (BioRad) at a final concentration of 1x, 400 nM of each of the forward and reverse primers, as described by Kim *et al.* [28], and 3 μL of genomic DNA. The final volume was made up with PCR grade water (Roche Diagnostics Limited, West Sussex, UK).

Total DNA concentration in genomic DNA extracts was measured using the Qubit dsDNA HS Assay Kit with the Qubit 1.0 fluorometer (Invitrogen, Paisley, UK) according to the manufacturer’s instructions. A mass of >1 ng DNA per extract was required for the Nextera XT library preparation (Illumina, Little Chesterford, UK). DNA samples below 0.2 ng μL^−1^ were concentrated by evaporation (SpeedVac, Thermo Scientific, Loughborough, UK), then resuspended in ultrapure water (Illumina) to 0.2 ng μL^−1^ for subsequent library preparation.

Specific quantification of *Cryptosporidium* DNA was performed by qPCR of the 18S rRNA gene using a protocol modified from Hadfield et al., [22]; only primers CRU18SF and CRU18SR (Integrated DNA Technologies, Glasgow, UK) at 900 nM, and the carboxyfluorescein-labelled minor groove binding TaqMan probe CRU18STM (Applied Biosystems, Warrington, UK) at 100 nM, were included. DNA concentration was estimated by comparing sample C_T_ with a standard curve created by testing linearised plasmid clones of the *C. hominis* 18S rRNA target region [22] serially diluted ten-fold in nuclease-free water, providing 10^1^ to 10^7^ copies per reaction. Each oocyst DNA extract was tested in triplicate and mean concentration calculated. C_T_ values were recorded and converted to oocyst equivalents mL^−1^ based on published evidence that each oocyst contains 20 copies of the 18S rRNA gene [29].

C_T_ values from initial suspensions and fully purified DNA were compared using a Mann–Whitney U test (Epi-info version 6, Centers for Disease Control and Prevention, USA). The reliability of the linear relationship between genome copies and mass of DNA in the final purified DNA extracts was measured by calculating the correlation coefficient (MS Excel).

### Next Generation Sequencing and Analysis

Barcoded paired-end libraries were created for each isolate with 1 ng of DNA using the Nextera XT DNA sample preparation kit (Version C protocol, Illumina). Amplicons >500 bp were size selected in the post-PCR purification steps. The resulting Nextera XT libraries were very low concentration, so after quantification by Qubit HS DNA assay, were pooled to give a 0.1 nM concentration and concentrated using a SpeedVac. In the initial phase, the Nextera XT libraries were sequenced on a MiSeq (Illumina) using 2 × 151 bp reads. In the main phase, libraries were sequenced on a HiSeq 2500 (Illumina) using a 2 × 151 bp rapid run.

Bioinformatics analysis was performed using CLC Genomics Workbench version 7.04 (CLC Bio, Aarhus, Denmark). Briefly, reads were paired, overlapping reads within a read pair merged, and trimmed based on a quality limit of 0.05, with a maximum of 2, ambiguities. To check sequencing coverage and depth, reads were subsequently mapped to the appropriate contemporaneous reference genomes, *C. hominis* TU502 v1 (Bioproject PRJNA 15585) and *C. parvum* Iowa II v1 (Bioproject PRJNA 15586) using CLC Genomics Workbench default settings (Masking mode = no masking, Mismatch cost = 2, Insertion cost = 3, Deletion cost = 3, Length fraction = 0.5, Similarity fraction = 0.8, Global alignment = No, Auto-detect paired distances = Yes, Non-specific match handling = Map randomly). Reads were also mapped to the *C. hominis* and *C. parvum* reference genomes using the Bowtie2 read aligner [13], with default settings, to the following quality parameters: high sequence identity and similar GC content (~30 %) to the reference genomes, a high-proportion of target DNA compared to background, and at least 30 X average sequencing depth.

*De novo* contig assembly for each isolate was performed using CLC Genomics Workbench (Mapping Mode = Create simple contig sequences, Automatic bubble size = Yes, Minimum contig length = 200 bp, Automatic word size = Yes, Perform scaffolding = Yes, Autodetect paired distances = Yes, Min distance = 180 bp, Max distance = 500 bp). The CLC *de novo* contig assemblies were improved using the post-assembly genome improvement protocol, PAGIT [12]. In brief, scaffolds were ordered using algorithm based automatic contiguation of assembled sequences (ABACAS) [30] against the *C. parvum* Iowa II reference genome; this genome comprises 18 contigs and mapping the assemblies to it generated 18 pseudomolecules that contained most of the assembled sequences, with typically 100 to 200 further much smaller scaffolds that could not be associated with the reference genome. Next, iterative mapping and assembly for gap elimination (IMAGE) [31] was used to close sequencing gaps in scaffolds of at least 500 bp. Smaller scaffolds were removed at this stage. Iterative Correction of Reference Nucleotide (ICORN) [32] was then used to correct single-base errors and small insertions and deletions of up to 3 bp. Lastly, the *C. parvum* Iowa II annotation was transferred onto the 18 pseudomolecules using rapid annotation transfer tool (RATT) [14]. The improvements made to the CLC assemblies using the PAGIT protocol were documented and are summarised in Table 4.

### Availability of supporting data

Whole genome sequencing data is deposited on the National Centre for Biotechnology Information (http://www.ncbi.nlm.nih.gov/) GenBank database as individual BioProjects (PRJNA253834, PRJNA253836, PRJNA253838-PRJNA253840, PRJNA253843, PRJNA253845-PRJNA253848) under the umbrella Bioproject PRJNA215218. 18S rRNA, *gp60*, *hsp70*, *COWP* and actin gene Sanger sequences are deposited under accession numbers KM085018-KM085027, KM012040-KM012055 and KP280061.”

## Abbreviations

ABACAS: Algorithm based automatic contiguation of assembled sequences
BLAST: Basic Local Alignment Search Tool
CsCl: Caesium chloride
ICORN: Iterative Correction of Reference Nucleotide
IMAGE: Iterative mapping and assembly for gap elimination
IMS: Immunomagnetic separation
NCBI: National Centre for Biotechnology Information
NGS: Next generation sequencing
PAGIT: Post-assembly genome improvement protocol
(q)PCR: (quantitative) Polymerase chain reaction
RATT: Rapid annotation transfer tool
WGA: Whole genome amplification
